# Supplementary material for: From sequence to enzyme mechanism using multi-label machine learning
Source: BMC Bioinformatics. 2014 May 19;15:150. doi: 10.1186/1471-2105-15-150 (PMC4229970; doi:10.1186/1471-2105-15-150)
Supplement: Additional file 2 — Java code of ml2db. Additional file ml2db_code.tar.gz contains the Java source code to run the multi-label machine learning experiments and save the results to database. The code’s Javadoc is included. [file 1471-2105-15-150-S2.zip › additional file 2/ml2db/ml2db/doc/main/ml2db/al/utility/globaldistance/package-use.html]

Uses of Package main.ml2db.al.utility.globaldistance


JavaScript is disabled on your browser.


- Overview
- Package
- Class
- Use
- Tree
- Deprecated
- Index
- Help

- Prev
- Next

- Frames
- No Frames

- All Classes

# Uses of Package main.ml2db.al.utility.globaldistance

- Packages that use main.ml2db.al.utility.globaldistance

  | Package | Description |
  | --- | --- |
  |  |  |
  | --- | --- |
  | main.ml2db |  |
  | main.ml2db.al.utility.globaldistance |  |
  | main.ml2db.al.utility.hybrid |  |
  | main.ml2db.weka |  |
  | test.ml2db.al.utility.globaldistance |  |
- Classes in main.ml2db.al.utility.globaldistance used by main.ml2db

  Class and Description

  DistancesCalculator

  Calculates the distance between two instances.
- Classes in main.ml2db.al.utility.globaldistance used by main.ml2db.al.utility.globaldistance

  Class and Description

  DistancesCalculator

  Calculates the distance between two instances.

  DistancesStorage

  Stores the distance between couples of instances to save computational time.

  GlobalDistanceMetric

  Positioning metric: a metric that considers positively if an instance is
  close to other unlabelled instances and distant from other labelled
  instances.

  GlobalPositionStore

  Stores (and updates) a map containing as key one (kind of/set of attributes)
  instance and as value the number representing the distance between the
  instance and a set of other labelled or unlabelled instances.
- Classes in main.ml2db.al.utility.globaldistance used by main.ml2db.al.utility.hybrid

  Class and Description

  GlobalDistanceMetric

  Positioning metric: a metric that considers positively if an instance is
  close to other unlabelled instances and distant from other labelled
  instances.
- Classes in main.ml2db.al.utility.globaldistance used by main.ml2db.weka

  Class and Description

  DistancesStorage

  Stores the distance between couples of instances to save computational time.
- Classes in main.ml2db.al.utility.globaldistance used by test.ml2db.al.utility.globaldistance

  Class and Description

  DistancesCalculator

  Calculates the distance between two instances.

  DistancesStorage

  Stores the distance between couples of instances to save computational time.

  GlobalDistanceMetric

  Positioning metric: a metric that considers positively if an instance is
  close to other unlabelled instances and distant from other labelled
  instances.

  GlobalPositionStore

  Stores (and updates) a map containing as key one (kind of/set of attributes)
  instance and as value the number representing the distance between the
  instance and a set of other labelled or unlabelled instances.

- Overview
- Package
- Class
- Use
- Tree
- Deprecated
- Index
- Help

- Prev
- Next

- Frames
- No Frames

- All Classes
